# The Lucy Thermal Emission Spectrometer (L’TES) Instrument

**DOI:** 10.1007/s11214-023-01029-y

**Published:** 2023-12-19

**Authors:** P. R. Christensen, V. E. Hamilton, G. L. Mehall, S. Anwar, H. Bowles, S. Chase, Z. Farkas, T. Fisher, A. Holmes, I. Kubik, I. Lazbin, W. O’Donnell, C. Ortiz, D. Pelham, S. Rogers, K. Shamordola, T. Tourville, R. Woodward

**Affiliations:** 1 School of Earth and Space Exploration, Arizona State University, Tempe, AZ USA; 2 Southwest Research Institute, Boulder, CO USA; 3 AZ Space Technologies, Gilbert, AZ USA

**Keywords:** Thermal emission spectrometer, Asteroid, Trojan, Lucy

## Abstract

The Lucy Thermal Emission Spectrometer (L’TES) will provide remote measurements of the thermophysical properties of the Trojan asteroids studied by the Lucy mission. L’TES is build-to-print hardware copy of the OTES instrument flown on OSIRIS-REx. It is a Fourier Transform spectrometer covering the spectral range 5.71–100 μm (1750–100 cm^−1^) with spectral sampling intervals of 8.64, 17.3, and 34.6 cm^−1^ and a 7.3-mrad field of view. The L’TES telescope is a 15.2-cm diameter Cassegrain telescope that feeds a flat-plate Michelson moving mirror mounted on a linear voice-coil motor assembly to a single uncooled deuterated l-alanine doped triglycine sulfate (DLATGS) pyroelectric detector. A significant firmware change from OTES is the ability to acquire interferograms of different length and spectral resolution with acquisition times of 0.5, 1, and 2 seconds. A single ∼0.851 μm laser diode is used in a metrology interferometer to provide precise moving mirror control and IR sampling at 772 Hz. The beamsplitter is a 38-mm diameter, 1-mm thick chemical vapor deposited diamond with an antireflection microstructure to minimize surface reflection. An internal calibration cone blackbody target, together with observations of space, provides radiometric calibration. The radiometric precision in a single spectrum is ≤2.2 × 10^−8^ W cm^−2^ sr^−1^ /cm^−1^ between 300 and 1350 cm^−1^. The absolute temperature error is <2 K for scene temperatures >75 K. The overall L’TES envelope size is 37.6 × 29.0 × 30.4 cm, and the mass is 6.47 kg. The power consumption is 12.6 W average. L’TES was developed by Arizona State University with AZ Space Technologies developing the electronics. L’TES was integrated, tested, and radiometrically calibrated on the Arizona State University campus in Tempe, AZ. Initial data from space have verified the instrument’s radiometric and spatial performance.

## Introduction

The Lucy Thermal Emission Spectrometer (L’TES) instrument will aid in the characterization of the Trojan asteroids observed by the Lucy mission (Levison et al. 2021; Olkin et al. 2021) through the determination of their thermophysical properties. The primary science objective of the L’TES investigation is to determine the regolith physical properties (primarily grain size) using daytime and nighttime temperature measurements. This objective is addressed using thermal infrared spectral observations between 1750 and 100 cm^−1^ (5.7 to 100 μm). The L’TES spectral range, spectral sampling, radiometric accuracy, and spatial resolution provide high-quality, mid-infrared spectra at sufficient ground resolution to investigate the physical properties of each of the Trojan asteroids that Lucy will visit – Eurybates, Leucus, Orus, Patroclus and Menoetius, which orbit each other, and Polymele. The L’TES calibrated radiance spectra will be converted to surface temperature(s) by fitting mixtures of Planck blackbody functions, with specified emissivity maxima (typically 0.97-1.0). Thermal inertia will be determined by fitting thermal models to the diurnal variation of the derived surface temperature(s) (e.g. Christensen et al. 2001; Kieffer 2013; Emery et al. 2014; Rozitis et al. 2020)

In this paper we present a description of the L’TES as-built instrument, calibration methods, and system performance. We also describe the basic instrument operational strategy, data processing methodology, and the plans for archiving the data through the Planetary Data System.

## Instrument Description

### Instrument Overview

The L’TES instrument continues the progression of infrared spectrometers and multi-spectral imagers developed by Arizona State University (ASU) for use on an array of planetary missions, including the Mars Observer (MO) and Mars Global Surveyor (MGS) Thermal Emission Spectrometer (TES) instruments (Christensen et al. 1992, 2001), the two Mars Exploration Rover (MER) Miniature TES (Mini-TES) instruments (Christensen et al. 2003), the Mars Odyssey Thermal Emission Imaging System (THEMIS) instrument (Christensen et al. 2004), the OSIRIS-REx OTES (Christensen et al. 2018), and the Emirates Mars Mission Infrared Spectrometer (EMIRS) (Edwards et al. 2021). A key feature of these instruments is that all have used uncooled bolometric detectors, which has allowed them to extend to longer wavelengths and be less complex with lower mass and cost than instruments using cooled photon detectors. This long-wave performance is essential for the measuring the Trojan asteroid temperatures, which are expected to be in the 60-120 K range.

L’TES is a Fourier-transform interferometer that collects hyperspectral thermal infrared data over the spectral range from 1750 to 100 cm^−1^ (5.7 to >100 μm) in a single detector. Unlike its predecessor, OTES, L’TES has selectable spectral sampling at 8.64 cm^−1^, 17.3 cm^−1^, and 34.6 cm^−1^. The three spectral samplings were chosen to allow short (0.5 sec and 1 sec), lower-spectral-resolution scans to be completed on the asteroid night sides where spatial sampling is more important than spectral resolution at the very low expected temperatures. L’TES has a 7.3-mrad full-width, half-maximum (FWHM) instantaneous field of view (IFOV) that allows nightside and dayside data to be collected during each flyby. L’TES is a build-to-print mechanical copy of OTES but has redesigned electronics from a different vendor than OTES.

The primary measurement requirement is to obtain spectra with sufficient absolute radiometric accuracy to allow the thermal inertia to be determined with sufficient accuracy to characterize the surface materials. L’TES will acquire full-disk integrated spectra during the approach mission phase (Levison et al. 2021; Olkin et al. 2021) and localized spatial information that will vary in resolution from ∼3 to 9 km at closest approach during the different flybys (Olkin et al. 2021). Because no single surface will be observed at different times of day, the L’TES diurnal observations taken of different locations will be combined to provide an estimate of the average asteroid thermal inertia.

### L’TES Measurement Requirements

#### Thermal Inertia

**Fig. 1:**
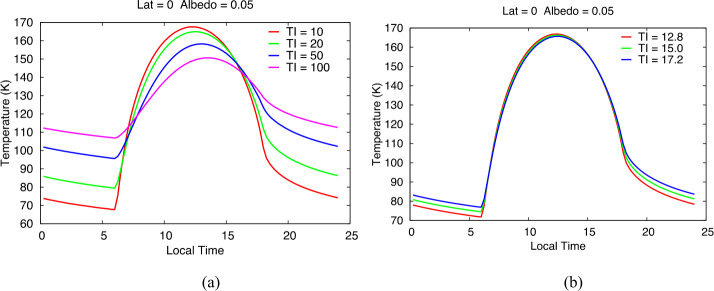
Modeled Trojan asteroid surface temperatures at the equator. **(a)** A representative range of expected thermal inertia values with a typical albedo value of 0.05. **(b)** Surface temperatures for a nominal thermal inertia of 15 SI and values of thermal inertia 15% higher (17.2 SI) and 15% lower (12.8 SI). These models were used to determine the L’TES absolute temperature requirement (2 K) necessary to determine the thermal inertia to ±15%

The L’TES thermal inertia requirement is to determine the surface thermal inertia to ±15%. Figure 1a shows modeled diurnal temperature variations for expected Trojan thermal inertias of 10 to 100 J m^−2^ K^−1^ sec^−1/2^ (hereafter referred to as SI) at the equator with an albedo of 0.05 using the krc thermal model (Kieffer 2013). Figure 1b gives the modeled diurnal temperatures for a surface with an inertia of 15 SI, together with models for 15% higher (17.2 SI) and 15% lower (12.8 SI) thermal inertias. Nighttime temperature differences between these models are ±2 K with the coldest temperature of ∼75 K occurring just prior to sunrise. These models result in an absolute temperature measurement accuracy requirement of 2 K for temperatures ≥75 K.

### L’TES Design

**Fig. 2:**
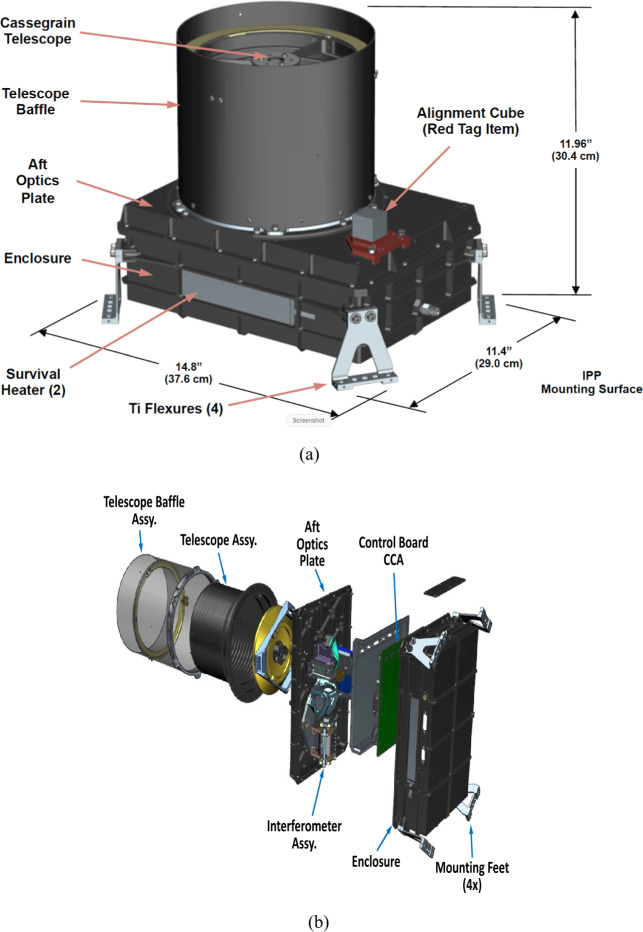
The L’TES CAD model. **(a)** The assembled instrument with dimensions. **(b)** L’TES, in an exploded view, showing the major elements of the L’TES modular design

**Fig. 3:**
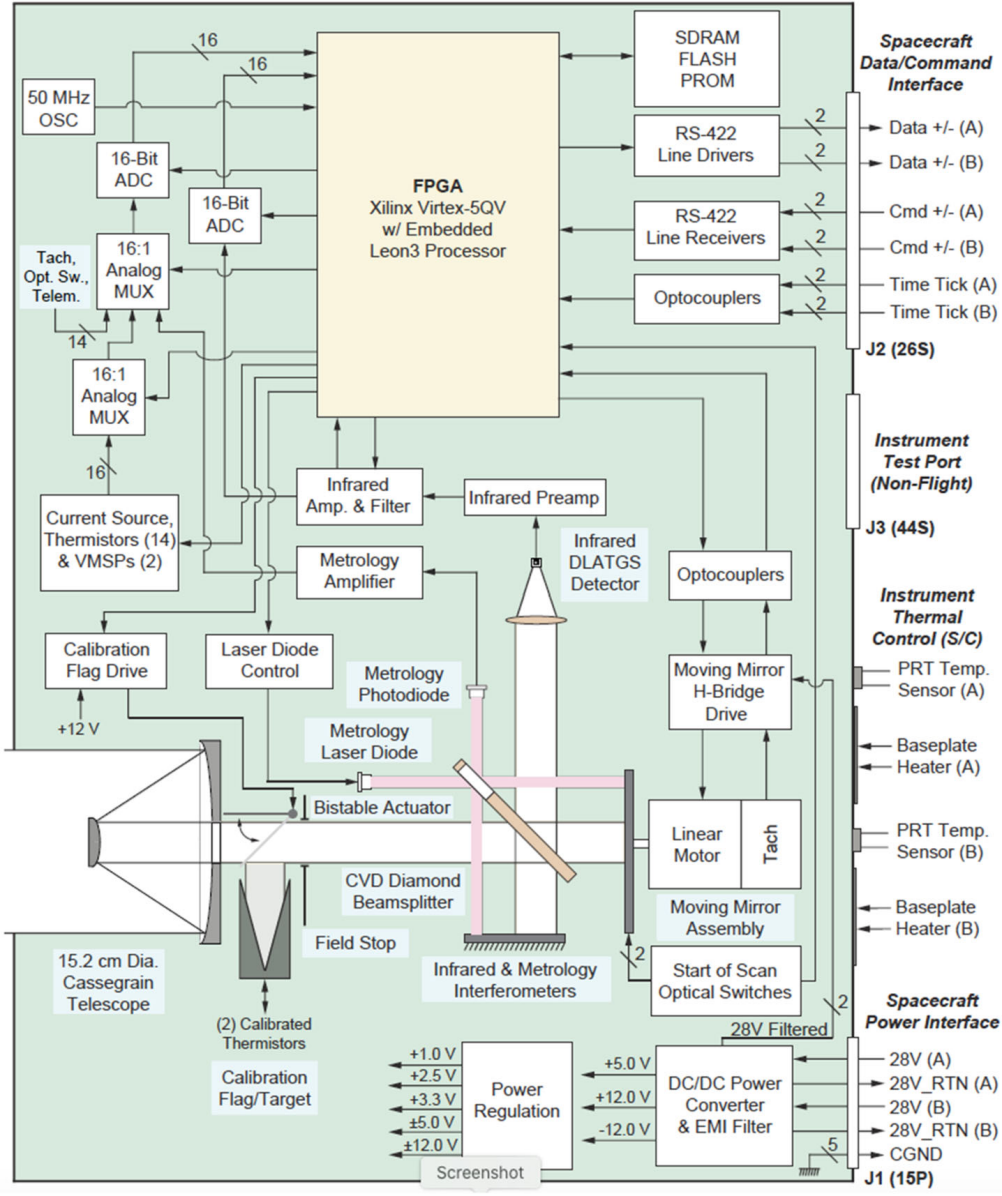
The L’TES block diagram, showing the key functional elements and the instrument-to-spacecraft interfaces

L’TES is a mechanical built-to-print copy of the OTES instrument (Christensen et al. 2018). The interferometer, detector, telescope, internal calibration assembly, and housing are identical to OTES. The only mechanical difference is a reduction in the length of the stray light baffle (“sunshade) that is appropriate for the geometric orientation of observations acquired at Jupiter’s orbit (Fig. 2). The only new development was the incorporation of electronics developed by a different vendor (AZ Space Technologies) than developed the OTES electronics. The L’TES electronics are based on those developed by AZ Space Technologies for the EMIRS instrument (Edwards et al. 2021) and incorporate enhancements to the digital servomechanism for the interferometer moving mirror that improved its performance, provided selectable moving mirror travel, and accommodated minor electronic parts obsolescence. The L’TES functional block diagram is shown in Fig. 3 and the instrument properties are summarized in Table 1.

**Table 1:** L’TES as-built instrument parameters

Parameter	Value
Spectral range	1750 to 100 cm^−1^ (5.71–100 μm)
Spectral sampling	8.64 cm^−1^, 17.28 cm^−1^, and 34.56 cm^−1^
Telescope aperture	15.2 cm
f#	3.91
Field of view (FWHM)	7.31 mrad in azimuth and 7.32 mrad in elevation
Detector	Uncooled deuterated l-alanine doped triglycine sulfate (DLATGS) pyroelectric
Detector D*	1.2 10^9^ cm Hz^1/2^ W^−1^ at 10 Hz, 22 °C
NESR (2-sec scan)	1.6 × 10^−8^ W cm^−2^ str^−1^/cm^−1^ from 300 to 1300 cm^−1^
Cycle times per measurement	2, 1, 0.5 s, including scan reversal time
Metrology laser self-apodized wavelength (25 °C)	0.851 μm
Michelson mirror travel	±0.281 mm; ±0.141 mm; ±0.070 mm
Michelson mirror velocity	0.319 mm/s
Sampling frequency	772 Hz
Number of bits per sample	16
Number of samples per interferogram—Filled	1360, 680, 340
Nominal data volume per 2 s scan	data: 21,760 bits; telemetry: 200 bits
In-flight calibration	Two-point; internal calibration blackbody and space
Cal blackbody emissivity	0.98 ±0.005
Thermal requirements	Operation in-spec: −10 °C to 30 °C
	Flight allowable operational range: −10 °C to +40 °C
	Non-operational protoflight survival range: −30 °C to +55 °C
Solar protection	Cal mirror in stowed position
Mass	6.47 kg
Power	12.6 W average; 18.0 W peak
Dimensions	37.6 × 29.0 × 30.4 cm

#### Opto/Mechanical

**Fig. 4:**
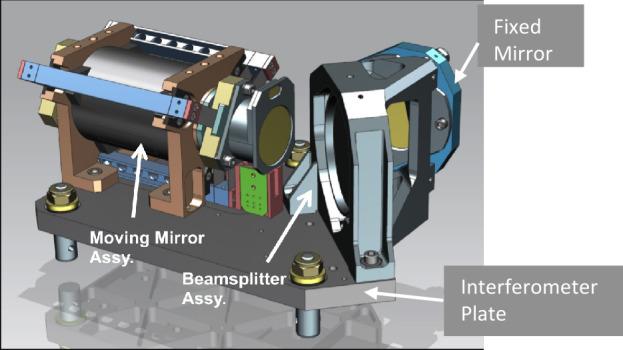
The L’TES CAD model showing the interferometer layout on the aft optics plate

The L’TES optical system uses a compact Cassegrain telescope design with a 15.2-cm diameter f/3.91 Ritchey-Chretien telescope that directs the incoming energy through a collimating off-axis parabolic mirror to create an optical beam with an afocal ratio of eight. This beam passes to the flat-plate interferometer moving mirror mounted on a voice-coil motor assembly (Fig. 4). The beam radiance passes through a CVD diamond beamsplitter installed in a radial three-point mount. Approximately half of the radiance is reflected to a fixed mirror, the other half is transmitted to the moving mirror, which travels ±0.281 mm to achieve the nominal 8.64 cm^−1^ spectral sampling. Selectable travel distances produce the additional spectral samplings of 17.3 cm^−1^ and 34.6 cm^−1^. The two optical beams recombine at the beamsplitter, producing the interference that determines the spectral distribution of the scene radiance, and travel to a parabolic mirror that images the beam onto a single on-axis detector element with a CVD diamond lens. A single monochromatic ULM VCSEL laser diode with a wavelength of 0.851 μm feeds a fringe counting metrology interferometer that uses the same moving and fixed mirrors and beamsplitter as used in the IR signal chain. The metrology interferometer is used to precisely control the velocity, track the position of the moving mirror, and trigger the sampling of the IR signal. The laser diode provides four times oversampling of the shortest scene bandpass wavelength. All mirror surfaces are diamond-turned and gold-coated, with reflectances of >0.99 across the L’TES spectral range. All of the L’TES machined components are aluminum for light weight and strength while meeting the launch load and pyro shock requirements. The use of baffles around the telescope housing and secondary mirror, and the use of diffuse black Z306 paint around the optics and within the cavity, minimize stray light effects.

L’TES utilizes a 38-mm-diameter, 1-mm-thick CVD diamond beamsplitter substrate. This diamond was fabricated by Diamond Materials and is installed in a radial three-point mount that provides precise alignment over the −10 °C to +30 °C operation-in-specification range, maintains mechanical integrity over the −30 °C to +55 °C nonoperational protoflight survival range, and accommodates the low thermal expansion coefficient and high heat conductivity of diamond. An antireflection microstructure (ARM) developed by TelAztec increases the transmission of raw diamond by ∼25% from 7 to >25 μm. Because light passes through the beamsplitter twice, this improvement represents an 50% increase in beamsplitter throughput. The low dispersion properties of diamond allow a single substrate to be used without the need for a compensator component, which would have increased the number of surface reflections and reduced the instrument throughput. The IR beamsplitting function is provided by a Ge beam-dividing coating. A parabolic mirror reimages the optical pupil onto an on-axis thermal IR detector that has a small (4.4 mm) CVD diamond lens also fabricated by Diamond Materials.

The L’TES internal calibration blackbody is a 1-cm-diameter, 15° half-angle cone that has two precision thermistors installed in its walls. These thermistors were calibrated to an absolute accuracy of 0.1 K, and the interior surface was painted with PT-401 paint resulting in a derived emissivity of the internal cal target of >0.99. The energy from this target reflects off a gold-plated mirror toward the interferometer. This cal mirror rotates 60° from its stowed position to reflect the radiance from the blackbody to the interferometer. The cal mirror is not instrumented directly, but it is stowed in the cal mirror cavity, whose temperature is determined from redundant, precision thermistors. (Christensen et al. 2018).

#### Detector

L’TES uses an uncooled deuterated l-alanine doped triglycine sulfate (DLATGS) pyroelectric detector fabricated by Leonardo Electronics (formerly Selex-Galileo). The measured D*, including the diamond lens, is 1.2 × 10^9^ cm⋅Hz^1/2^/W at 10 Hz, 22 °C. The detector has a bias voltage applied to the Field Effect Transistor (FET) to ensure it remains properly poled. An identical detector was used on the OTES instrument. Preamplification and front-end filtering are performed on the preamplifier circuit board to amplify the signal and to AC couple the detector output to block high-frequency oscillations.

#### Electronics

A ±12-volt regulator supplies power to the detector and preamplifier electronics. The spectrometer circuit board performs the bulk of the analog electronics processing. L’TES command, control, and data flow tasks are controlled by logic in the command-and-control field programmable gate array (FPGA). The interface electronics parse out the instrument commands that control L’TES hardware functions. The flow of the digital interferometer data is controlled by additional logic in the command-and-control board FPGA. After each interferometer scan, the 16-bit interferogram data and 16-bit telemetry data are moved from the analog to digital converter (ADC) to the input memory buffer on a 16-bit parallel data bus. The 16-bit data are then serialized for transfer to the spacecraft (S/C) via RS-422 interface. The analog multiplexer, digital serializer, and data formatting logic are included in the command-and-control FPGA. The DC power converter accepts 24 to 34 volts unregulated input voltage and supplies +3.3, ±5 and ±15 volts regulated output voltage.

The L’TES timing sequencing electronics are implemented in the command-and-control board FPGA. These electronics generate the timing waveforms necessary to control and synchronize instrument operation and provide the control and synchronization of the amplification, track/hold, multiplexing, and analog to digital conversion of the detector and telemetry signals. They also control and synchronize the interferometer servo electronics with the data acquisitions. The timing sequencing electronics include the fringe delay electronics that are used to correct the sampling error due to the phase delays between the fringe and IR analog channels. All clocks in the timing sequencer are generated from the master clock crystal oscillator that operates at a frequency of 20 MHz and is synced to the spacecraft clock. The L’TES interferometer servo electronics were completely redesigned from previous instruments to use a digital servo drive running in the FPGA. This digital servo receives scan timing clocks from the timing sequencer electronics and the fringe clock from the fringe detection electronics. The motor control logic uses these clocks to synchronize the mirror movement with the spectrometer data acquisitions. The moving mirror uses a direct drive BEI Kimco linear motor with tachometer feedback. The moving mirror tachometer signal is returned to the interferometer control electronics to allow active feedback control of the actuator.

The digital servo control loop is implemented using a custom processor core with a double precision Field Programmable Unit (FPU) that runs inside an FPGA mounted on the command control board. The control loop uses digitized metrology fringe, tachometer, and end-of-travel optical switch as inputs; performs fringe data processing, feedback compensation, sample timing, and command/telemetry functions; and outputs the motor command to the D/A converter controlling the voice-coil driver circuit for the interferometer moving mirror.

High sample frequency of the fringe signal (20 kHz) enables the control loop to estimate the servo velocity at a higher frequency than would be achievable by only monitoring the metrology interferometer zero crossings. A rate estimation algorithm inside the servo controller uses the sinusoid model of the metrology fringe to derive a servo rate estimate at a 2-kHz rate. This model is also used to predict the appropriate times to sample the interferometer detector. The detector samples are based on estimated fringe signal zero crossings and are delayed to compensate for the phase delay of the Bessel filter in the detector electronics. An optical switch is used near the end of travel to measure the absolute position and control the servo motion so that it remains centered about the zero-path difference (ZPD) position. The control algorithm includes adjustable optical switch thresholds so that the scan pattern can be centered without physically moving the optical switch assembly.

There is a great deal of flexibility in the design and adjustment of the digital servo. Controller gains and limits, as well as some other configuration options, such as parameters controlling scan timing, can be changed via commands while the controller is executing out of SRAM. Also, because the entire algorithm is stored in nonvolatile memory, it can be modified without changes to hardware.

The flow of the digital interferometer data is controlled by additional logic in the command-and-control board FPGA. After each interferometer scan, the 16-bit interferogram data and 16-bit telemetry data are moved from the A/D to the input memory buffer on a 16-bit parallel data bus.

#### Thermal Design

L’TES is a thermal instrument that requires thermal stability of <0.1 °C per minute in order to maintain calibration between periodic calibration observations. This stability is achieved by conductively isolating L’TES from the spacecraft to prevent rapid thermal transients, while radiatively coupling L’TES to the spacecraft to provide a long-term thermal sink. Replacement heaters and thermal blanketing are used to maintain the instrument within its flight allowable temperature limits. L’TES will operate within specification over a temperature range of −10 to +30 °C, operate from −10 to +40 °C, and survive form −30 to +55 °C.

#### S/C Interface

The L’TES spectrometer provides data to the spacecraft across two synchronous RS-422 serial lines at 57.6 Kbps during the 200-msec interferometer scan reversal period whenever L’TES is powered. Instrument commanding, including operation of the internal cal mirror and the selection of redundant components, is provided across two synchronous RS-422 serial lines at 57.6 Kbps. Synchronization of the interferometer is performed by L’TES using the spacecraft clock. The L’TES power interface uses redundant unregulated power lines to the input of the L’TES DC/DC converter. The unregulated input power can be from +24 to +34 VDC, with a nominal value of 28 VDC.

#### L’TES Radiation and Contamination Mitigation

L’TES is based on the OSIRIS-REx OTES and MGS TES instruments that were designed to survive 20 krad environments. It is estimated that the MGS TES exceeded this radiation dose in its 10-year life, with no detectable radiation damage effects. OTES has operated for >5 years in a radiation environment similar to that of Lucy with no detectable degradation in performance. L’TES was built following the Goddard Space Flight Center and Lucy Project assembly and test procedures to minimize any organic contamination carried on the instrument. Contamination on the instrument would have to be extreme (>10 s of μm) to degrade the performance based on experience with Mini-TES in the dusty surface environment of Mars (Ruff et al. 2011). Analysis and monitoring of witness samples through the L’TES development at ASU demonstrate that L’TES was delivered with a cleanliness level D per NASA CR 4740.

#### L’TES Operational Modes

L’TES begins collecting telemetry data when power is applied. Upon receipt of the data collection command, L’TES collects and transmits interferograms to the spacecraft continuously when operational. L’TES will begin acquiring data of each Trojan several hours prior to closest approach when the asteroid fills ∼1% of the L’TES field of view and continue for several hours after closest approach. Spatially resolved observations will be collected during the flyby through two-axis scanning of the instrument pointing platform (IPP) to point L’TES at the desired locations. L’TES has two operational modes – data collection and standby. In data collection mode L’TES collects one interferogram every 0.5, 1, or 2 seconds; in standby mode no interferogram data are collected, but telemetry data are collected once every two seconds. Instrument commands are used to insert the internal cal flag mirror into the optical path, to select the scan period, and to switch between the redundant start-of-scan optical switches.

## Calibration Methodology

### Calibrated Radiance

The L’TES calibration approach is identical to that of OTES and is described in detail in Christensen et al. (2018). In summary, the L’TES interferometer measures the difference in the modulated spectral radiance ($I$; in W cm^−2^ sr^−1^ /cm^−1^) coming alternatively from the external scene and from the detector itself as the interferometer moving mirror translates. The external radiance is the sum of the radiance from the scene that enters the aperture, as limited by the field stop ($I_{\mathit{scene}}$), the radiance from the optics themselves ($I_{\mathit{optics}}$), as well as any radiance from the field stop and the interior of the instrument that is modulated through the interferometer ($I_{\mathit{instrument}}$). The radiance from the interior of the instrument that does not pass through the interferometer is not modulated and is a DC term in the interferogram that will be absent from the transformed spectrum. The difference between the modulated external radiance and that from the detector ($I_{\mathit{detector}}$) is the signal measured by the detector.

The optics radiance ($I_{\mathit{optics}}$) can be divided into the emitted radiance from the fore optics ($I_{\mathit{fore}}$; i.e. the telescope) and the aft optics ($I_{\mathit{aft}}$; collimating, transfer, and detector focusing optics). The aft optics radiance does not pass through the interferometer and is not modulated, so only the fore optics radiance becomes a component of the interferogram signal. The radiance from the instrument ($I_{\mathit{instrument}}$) includes the radiance emitted from the field stop, which is modulated through the interferometer. In practice, however, this modulated radiance is indistinguishable from the detector radiance; this radiance will be included in the detector radiance in the subsequent discussion.

Using these definitions, the electrical signal ($V$) produced by the interferometer is given by:

1 $$ V= \left ( I_{scene} + I_{fore} - I_{detector} \right ) *IRF $$

where *IRF*, in units of Volt/(W cm^−2^ /cm^−1^ sr^−1^), is the instrument response function that converts the optical input to electrical output. Observations of two calibration targets allow the $I$ and *IRF* terms to be determined and used to determine the scene radiance.

The radiance from the scene is given by:

2 $$ I_{scene}=\varepsilon_{scene}B_{scene}\tau_{optics} $$

where:

$\epsilon _{\mathit{scene}}=$ emissivity of the scene

$B_{\mathit{scene}}=$ Planck emission from the scene

$\tau _{\mathit{optics}}=$ throughput of the full optics

The determination of the desired scene radiance ($I_{\mathit{scene}}$) from Eq. (1) requires knowledge of the two unknown terms ($I_{\mathit{fore}}$ – $I_{\mathit{detector}} $) and the *IRF*, which in turn requires the observation of two calibration targets of known radiance. As with OTES, the L’TES internal calibration blackbody is located behind the telescope and the *IRF* cannot be determined simply from observations of the two calibration targets because the same optical path is not viewed in the scene, space, and cal blackbody observations, and the $I_{\mathit{fore}}$ term does not cancel as it does in the two-target, full-optics method. In this case the fore optics throughput and emission must be estimated. The uncertainties in these estimates result in errors in the scene radiance, the magnitude of which can be investigated by modeling the scene radiance as the optical throughput and emission are varied over their uncertainties (see Sects. 3.2 and 6.7).

As derived in Christensen et al. (2018), the scene radiance ($\epsilon $_scene_B_scene_) is given by:

3 $$\begin{aligned} &\varepsilon_{scene}B_{scene} \\ &\quad = \left(\frac{V_{scene}-V_{space}}{V_{cal}-V_{space}}\right) \\ &\qquad {}\times \left(\frac{\varepsilon_{cal}B_{cal}\rho_{flag}+\varepsilon_{flag}B_{flag}- \left(\varepsilon_{primary}B_{primary}\rho_{secondary}+\varepsilon_{secondary}B_{secondary}\right)}{\tau_{fore}} \right. \\ &\qquad \left.{}-\varepsilon_{space}B_{space}\right)+\varepsilon_{space}B_{space} \end{aligned}$$

where *cal* is the internal calibration target, *flag* is the gold coated flag that is inserted into the beam path to observe the cal target, *space* refers to the space calibration observation, and *primary* and *secondary* refer to the two Cassegrain telescope mirrors.

### Absolute Calibration

The absolute accuracy of the scene radiance is a function of the uncertainty in the knowledge of each of the terms in Eq. (3). The uncertainty of the signal terms ($V$) is determined by the instrument noise of each measurement, whereas the uncertainty in the emissivity, reflectance, and temperature of the calibration target and mirrors is determined by the accuracy to which each of these terms can be measured. Averaging multiple observations can reduce the uncertainty due to instrument noise; however, the uncertainties in the remaining terms cannot be reduced and are the driving terms in the absolute accuracy. The uncertainties due to the emissivity, reflectance, and temperature of the cal target and fore optics were modeled by assessing the effects of the uncertainties in each of these parameters using Eq. (3). This equation has been applied in a Monte Carlo model to assess the absolute error in the scene radiance using: (1) the measured random noise errors in the signals ($V_{\mathit{scene}}$, $V_{\mathit{space}}$, and $V_{\mathit{cal}}$); (2) the uncertainty in the radiance from the cal and space targets ($B_{\mathit{cal}}$, and $B_{\mathit{space}}$) and the primary and secondary mirrors ($B_{\mathit{primary}}$, $B_{\mathit{secondary}}$) that are due to temperature errors in the measurement of each of these elements; and (3) the uncertainty in the emissivity of the calibration blackbody and the reflectivity of the primary and secondary mirrors and the calibration flag.

**Fig. 5:**
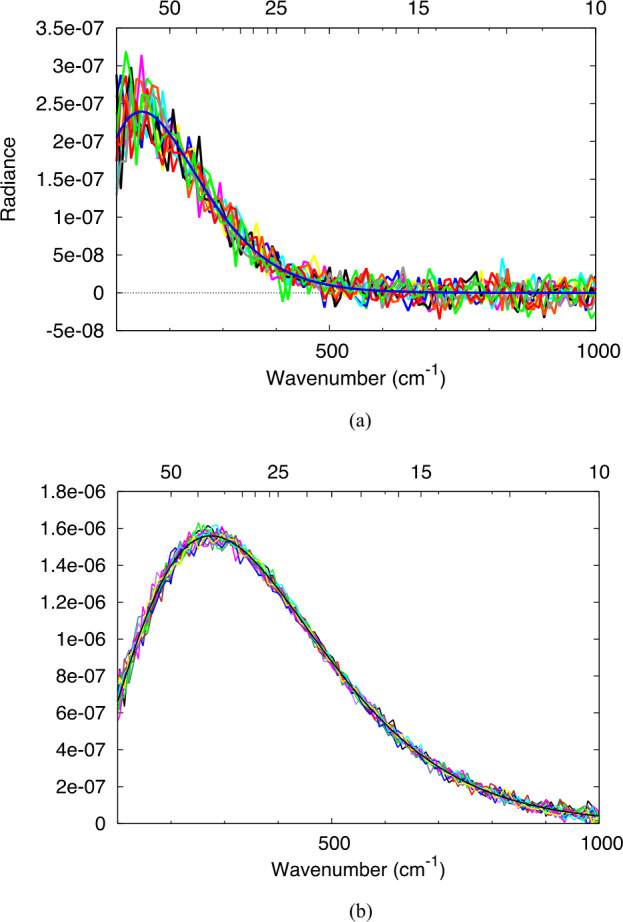
Modeled L’TES radiance spectra compute by applying random noise equivalent to the measured NESR versus wavenumber to a Planck function assuming an emissivity of unity. **(a)** Radiance from a surface at 75 K. **(b)** Radiance from a surface at 140 K. Upper scale is micrometers

The worst-case expected absolute error in the cal target temperature is 0.5 C and its emissivity is 0.005 (Christensen et al. 2018). These estimated errors were included in a Monte Carlo model that incorporated the measured L’TES flight instrument response function and instrument noise (noise equivalent spectral radiance; NESR) (see Sect. 6). The simulated L’TES spectra were converted into scene radiance that was then converted to brightness temperature at each wavenumber, assuming an emissivity of unity. The surface kinetic temperature, which is the desired parameter for temperature mapping, was determined by averaging the brightness temperature over a specified wavenumber range. The full spectral range was used for warm targets, whereas narrower low-wavenumber ranges were used at lower surface temperatures. As a result, the NESR begins to affect the accuracy of the derived surface kinetic temperature at temperatures below ∼125 K because fewer spectral channels are available to average the brightness temperature. This process is roughly equivalent to fitting a blackbody radiance curve to the measured radiance to provide a best-fit determination of the surface temperature. For the very low temperatures expected for the Trojan asteroids, the measurement noise becomes a significant contributor and dominates the temperature determination for scene temperatures below ∼120 K. Figure 5 demonstrates the effect of instrument noise at low temperatures for modeled spectra at scene temperatures of 75 and 140 K using the measured L’TES noise performance (Sect. 6.6).

**Fig. 6:**
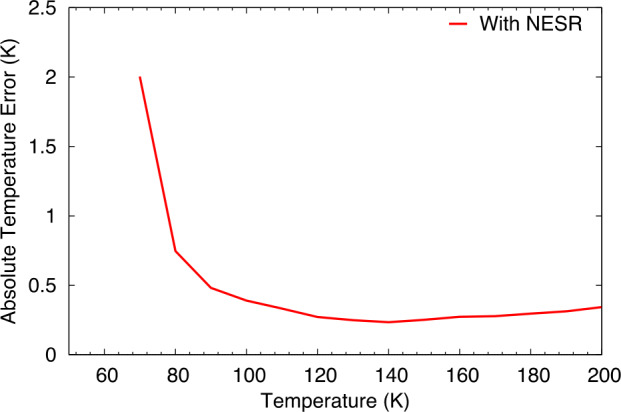
Modeled error in the derived surface temperature. The model incorporates a Monte Carlo analysis of the expected uncertainties in each of the calibration parameters (Eq. (3)) together with the measured NESR to compute a series of radiance spectra. The kinetic temperature was derived from these model spectra and the 1-sigma variation was determined and plotted versus scene temperature

The model was run for 10,000 random cases for surface temperatures from 70 to 400 K at 10 K intervals, and the standard deviation of the derived surface kinetic temperature was computed for each surface temperature (Fig. 6). These values represent the best estimate of the L’TES absolute temperature accuracy errors. As seen in Fig. 6 L’TES meets the absolute temperature requirement of ±2 K for surface temperatures ≥75 K. The measured absolute temperature performance using the results from the pre-launch test data are given in Sect. 6.

## Experimental Setup

### Test Equipment and Facilities

The L’TES instrument was assembled, tested, and calibrated on the Arizona State University Tempe campus in an ISO Class 7 (class 10,000) cleanroom in building ISTB4, with Class 6 (1,000) flow benches for component assembly (Christensen et al. 2018). L’TES calibration was done in both ambient and thermal vacuum conditions using test equipment that was calibrated to NIST standards on a routine basis. Bench-level testing of the L’TES instrument consisted of piece-part and system-level testing of each sub-assembly under ambient conditions, followed by system performance testing that was conducted before and after each environmental test and throughout thermal-vacuum testing. Ambient tests determined the instrument functional performance, the field-of-view definition and alignment, the out-of-field response (encircled energy), the spectrometer spectral range and sample position, and verified the command and data links. The thermal vacuum calibration testing determined the instrument response function in vacuum over the expected range of instrument temperature, the signal gain value, the emissivity and temperature of the internal calibration blackbody, and the radiometric precision and accuracy.

Throughout the integration and test phase an Instrument Functional Test (IFT) was performed using the L’TES ambient target and/or two calibration reference blackbody standards (Bench Checkout Units; BCU) (Christensen et al. 2018) to provide trending of performance throughout the test program. A Comprehensive Performance Test (CPT) was performed using the ambient and/or BCU targets and provided the baseline radiometric performance assessment for the instrument. This test was also performed before and after environmental tests. The L’TES optical field of view was characterized using an 8-inch-diameter f/7.5 off-axis collimator that was scanned in elevation and azimuth using computer-controlled actuators.

The L’TES thermal vacuum radiometric calibration was performed using the two BCU calibration targets that were placed inside the thermal vacuum chamber. The absolute temperature of these targets was determined using precision thermistors that were NIST-calibrated to ±0.1 °C. Their emissivities were determined to be 0.99897 ±0.0002 based on the spectral properties of the PT-401 paint and the analysis of a cone blackbody (Bedford 1988; Prokhorov et al. 2009). The temperature of the primary and secondary mirrors was determined using the NIST calibration of the flight thermistors mounted to these elements, and the reflectivity was determined using laboratory measurements of witness samples of the gold coatings that were applied to the optical surfaces.

L’TES was radiometrically calibrated in vacuum at instrument temperatures of −10, 0, 10, and 25 °C. Calibration tests were performed by viewing the two BCU targets, one set to a temperature of 85 K to simulate space observations and the second set to temperatures of 100, 150, 180, 210, 240, 270, 300, and 330 K to span the expected range of target surface temperatures. L’TES was installed in a LACO thermal vacuum chamber developed for the L’TES project in the Class 8 (100,000) cleanroom in ISTB4 on the ASU campus. L’TES was mounted on a metallic L-plate with thermostatically controlled strip heaters that simulated the instrument pointing platform deck for thermal balance and was used to control the instrument temperature during thermal vacuum testing and thermal cycling. A liquid-nitrogen-cooled plate was attached to the back of the mounting plate to provide a cooling source for the bracket/instrument. L’TES was configured in the chamber to allow its aperture to point between the two external BCU blackbody calibration targets using a vacuum-compatible rotary stage attached to the underside of the L-plate (Christensen et al. 2018). The instrument and mounting bracket were fully blanketed with flight-like Multi-Layer Insulation (MLI) thermal blankets to simulate the IPP thermal configuration. Ground Support Equipment (GSE) controlled the L’TES, L-plate, targets, and rotary stage via cabling through the chamber feedthroughs.

### Data Processing Software/Scripts

The L’TES data were processed using the open-source vector math software package called *davinci* that was developed at ASU for instrument data processing and has been used for calibration and processing of the TES, Mini-TES, THEMIS, OTES, and EMIRS data. The L’TES performance varies slightly between the forward and reverse moving mirror scan directions, so all processing is done separately for the two scan directions. The discrete Fourier transform (DFT) is performed on the interferograms, bad spectra resulting from chamber- or spacecraft-induced noise are identified and removed, and the internal and external calibration targets and scene target are identified using telemetry. Groups of calibration spectra are averaged and linearly interpolated over time and the radiometric calibration and data analysis are performed.

## L’TES Development

### Flight Instrument Development

The L’TES instrument began development in January 2017 with the selection of the Lucy mission for Phase B. The System Requirement Review (SRR) was held on Oct. 24, 2017. The Preliminary Design Review (PDR) was held on April 13, 2018, and the Critical Design Review (CDR) on March 19, 2019. L’TES was delivered to the spacecraft on December 13, 2020, with a total development time from SRR to delivery of 38 months. The most challenging aspect of the L’TES development program was the assembly, test, and calibration of the instrument during the height of the COVID-19 pandemic between January and December 2020. The L’TES team undertook stringent measures to reduce the risk of infection, including stringent use of mask and glove protocols, limiting the number of personnel in the assembly cleanroom at any time to three, separating assembly and inspection shifts into morning and afternoon operations, and holding technical interchange discussions via video conferencing. The most challenging technical issue was the alignment of the interferometer. The interferometer fixed and moving mirrors and the beamsplitter are mounted on a vibration isolated plate (Fig. 4). The fixed mirror is mounted to the beamsplitter housing on a three-point mount and aligned using nuts on the three threaded studs. These nuts are injection bonded following final alignment to prevent movement during launch. Following the initial L’TES thermal cycling, the interferometer alignment was observed to have degraded, as evidenced by a decrease in the IRF at short wavelengths. The spectral shape of this reduction was accurately modeled as a tilt in the fixed mirror relative to the beamsplitter housing. Subsequent analysis revealed that minor oxidation on the three studs and minor roughness on the nuts holding the fixed mirror resulted in a 13 arcsec tilt of the fixed mirror following initial heating. Once this first-time “settling” occurred, the fixed mirror exhibited the expected 2-3 arcsec tilt over the full operational temperature range from −10 to +40 °C. Through test it was determined that torquing, releasing, and re-torquing the fixed mirror mounting nuts significantly reduced the first-time shift. The original flight beamsplitter assembly, whose nuts had been bonded, was replaced with the spare assembly and testing continued. A second, related issue was a change in the alignment of the metrology laser over temperature that resulted in a variable laser signal with temperature. If the laser signal increased beyond the analog-to-digital saturation limit, then the servo control algorithm would not function properly. The tilt over temperature issue was corrected as discussed above, but as a precaution the metrology laser lens was redesigned to reduce its signal sensitivity to interferometer tilt.

**Fig. 7:**
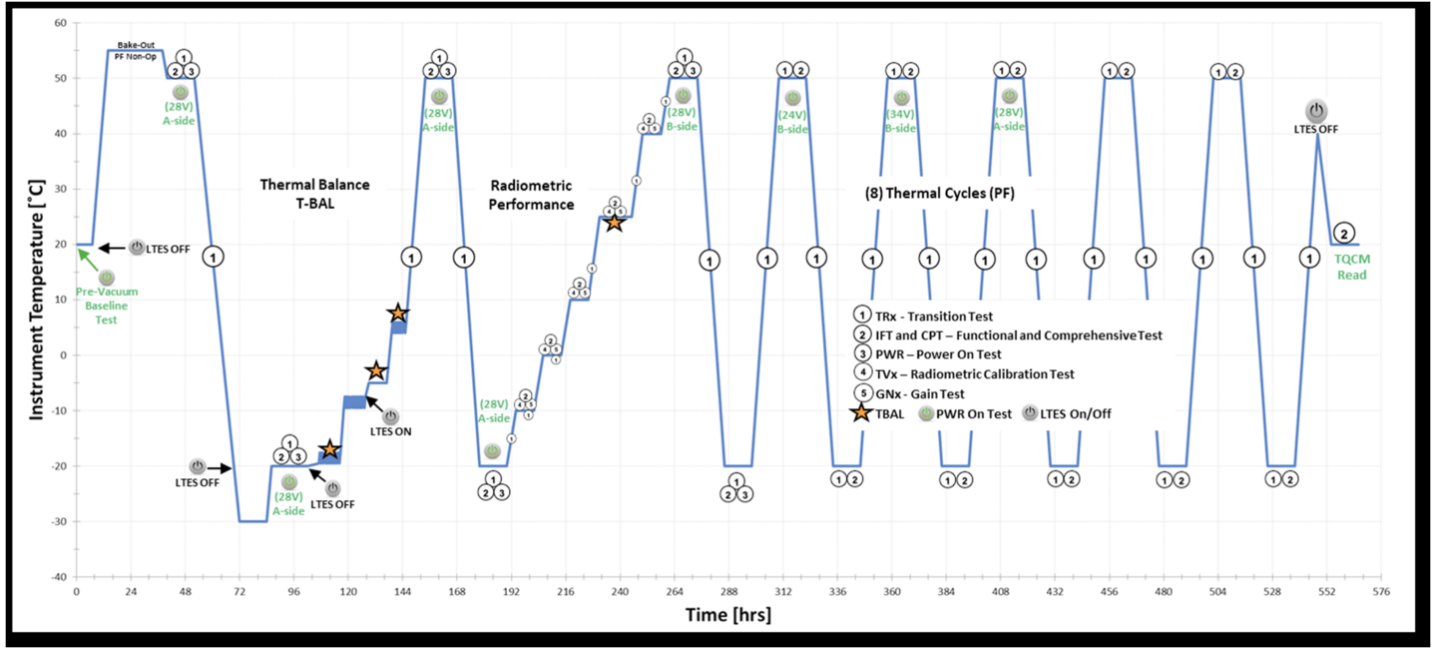
The L’TES thermal balance and thermal vacuum test timeline

L’TES underwent its final thermal vacuum testing from October 30 through November 23, 2020, with the radiometer calibration occurring between November 9 and 15th. These tests included radiometric calibration at multiple instrument temperatures, as well as eight thermal cycles from −30 to +50 °C (Fig. 7).

### Post-Delivery Testing

Following the delivery of the flight instrument to the spacecraft developer and flight system integrator, Lockheed Martin Corporation (LMC; Olkin et al. 2021), a noise source was observed during system-level testing. This noise was associated with the spacecraft inertial measurement units (IMU), which produce discrete vibrations in the 500–700 Hz range that have a magnitude of ∼5–10 milli-G at the L’TES spacecraft mounting surface. In its original configuration, the L’TES IR interferometer collected samples at 656 Hz. The servo control uses the signal from the metrology laser signal to predict when the laser signal will go to zero and when the IR signal should be sampled. The IMU-induced vibrations resulted in small, periodic jitter in the moving mirror position, and the zero-position of the mirror had slight (∼1% of the sampling distance) offsets. The magnitudes of these periodic offsets are small (0.01 × 1/2 × 0.849 μm or 0.004 μm), but they result in artificial sinusoids at specific frequencies that correspond to the difference in frequency between the L’TES 656 Hz sampling frequency and the IMU frequencies. Two of these beat frequencies occurred at ∼40 and 100 Hz, which is within the L’TES information band from 10 to 120 Hz, and resulted in spikes in the L’TES IR spectrum. A similar problem was observed on OTES, and the same solution used for OTES was implemented on L’TES. This solution involved increasing the servo velocity to 0.321 mm/s, giving a sampling frequency of 772 Hz, which moves the IMU frequency disturbances outside the L’TES information band. In-flight data from OTES confirm that this solution works very well. The values given in Table 1 correspond to the higher servo velocity that will be used in flight.

## Pre-Launch Calibration Results

### Gain

The L’TES gains were set to nominal values of 1×, and 2× using individual precision resistors. Gain 2× was set for the nominal instrument performance and expected space and target temperatures. A gain of 1× was included as a safety factor to accommodate unexpected instrument behaviors or asteroid characteristics. The actual gain values were determined using data acquired during the thermal vacuum testing at five instrument (detector) temperatures of −5.8, 3.4, 14.1, 27.5, and 51.8 °C. The method used the interferogram peak-to-peak (P-P) value, averaged for 90 spectra in each of the forward and reverse scan directions at each of the two gain states for each instrument temperature. The peak-to-peak values correspond to the ZPD position in the interferogram and have the highest signal-to-noise ratio, providing the best indicator of the true gain values. For each test the target temperature was ∼27 °C. The computed 2× gain was 1.973 ±0.00489 with no systematic temperature variation in the gain values. In normal operations all data within an observing sequence will be acquired using the high gain, so no correction for gain is required. If comparing data acquired with different gain settings, or if different gain settings are used (off-nominal case), then a gain correction of 1.973 should be applied.

### Field of View

**Fig. 8:**
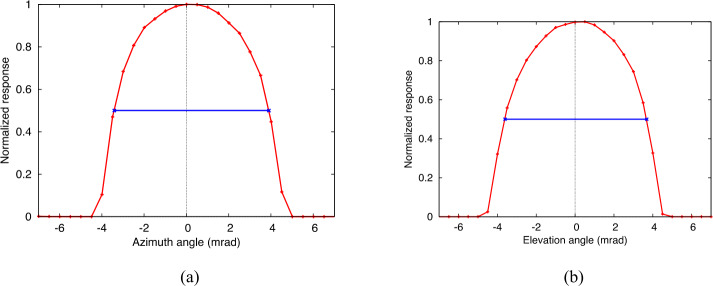
The L’TES field of view measured following environmental and thermal vacuum testing, and immediately prior to delivery to the spacecraft. **(a)** Azimuth. **(b)** Elevation

The L’TES field of view was determined in azimuth and elevation using a collimator viewing a glowbar target through 1-mrad-wide vertical or horizontal slits placed at the collimator focus (Christensen et al. 2018). The collimator was oriented parallel to the L’TES optical axis (0° azimuth, 0° elevation). A thermally stable shutter was used that was alternatively opened to view the glowbar/slit or closed to view the shutter to remove any thermal drift in the system. Twenty spectra were collected with the shutter open and 20 with the shutter closed at each azimuth or elevation step. The slit was moved at 0.5-mrad steps, starting and ending seven degrees outside of the nominal field of view. The analysis averaged the value of the peak-to-peak of the ZPD position of the interferogram to maximize the signal-to-noise ratio. The thermal drift was removed and the data were normalized to the minimum and maximum P-P values. The field of view results following the environmental testing and final pre-ship alignments are given in Fig. 8. The field of view, defined at the full-width, half-maximum (FWHM) point in the normalized data, is 7.31 mrad in azimuth and 7.32 mrad in elevation. The L’TES optical axis was measured relative to the L’TES alignment cube, and a transformation matrix for the optical axis relative to the spacecraft-mounting interface was provided to LMC for the L’TES spacecraft alignment and pointing determination.

### Out-of-Field Response

**Fig. 9:**
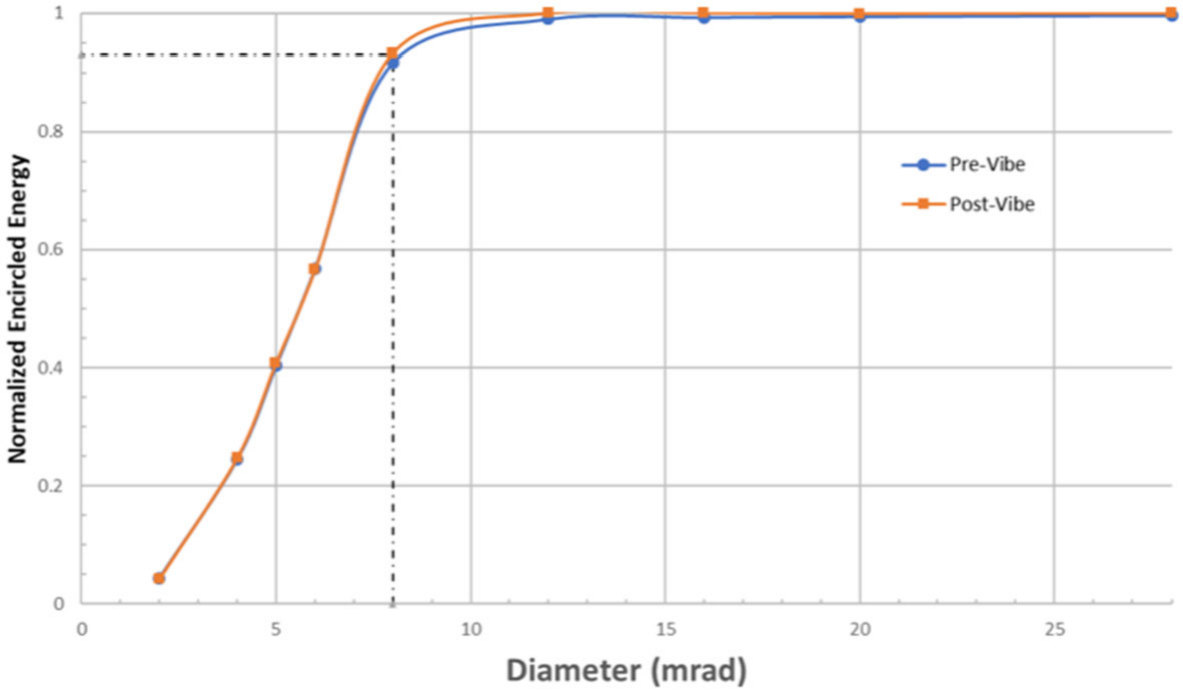
The L’TES encircled energy measured pre- and post-vibration testing and immediately prior to delivery to the spacecraft. The purple data point shows the requirement of ≥85% of the energy in an 8-mrad field

The L’TES out-of-field response was determined by measuring the encircled energy performance using the collimator with a set of fixed apertures with diameters of 2, 4, 6, 8, 12, 16, 20, 28, and 36 mrad that were placed at the collimator focus. The encircled energy test collected 30 spectra with the shutter open and 30 with the shutter closed at each azimuth or elevation step. The analysis used the averaged values of the peak-to-peak of the interferogram to maximize the signal-to-noise ratio. The thermal drift was removed, and the data were normalized to the minimum and maximum P-P values. The results are shown in Fig. 9 and demonstrate that L’TES easily exceeds its requirement for 85% of the measured energy coming from one geometric scene footprint of 8 mrad.

### Instrument Response Function

**Fig. 10:**
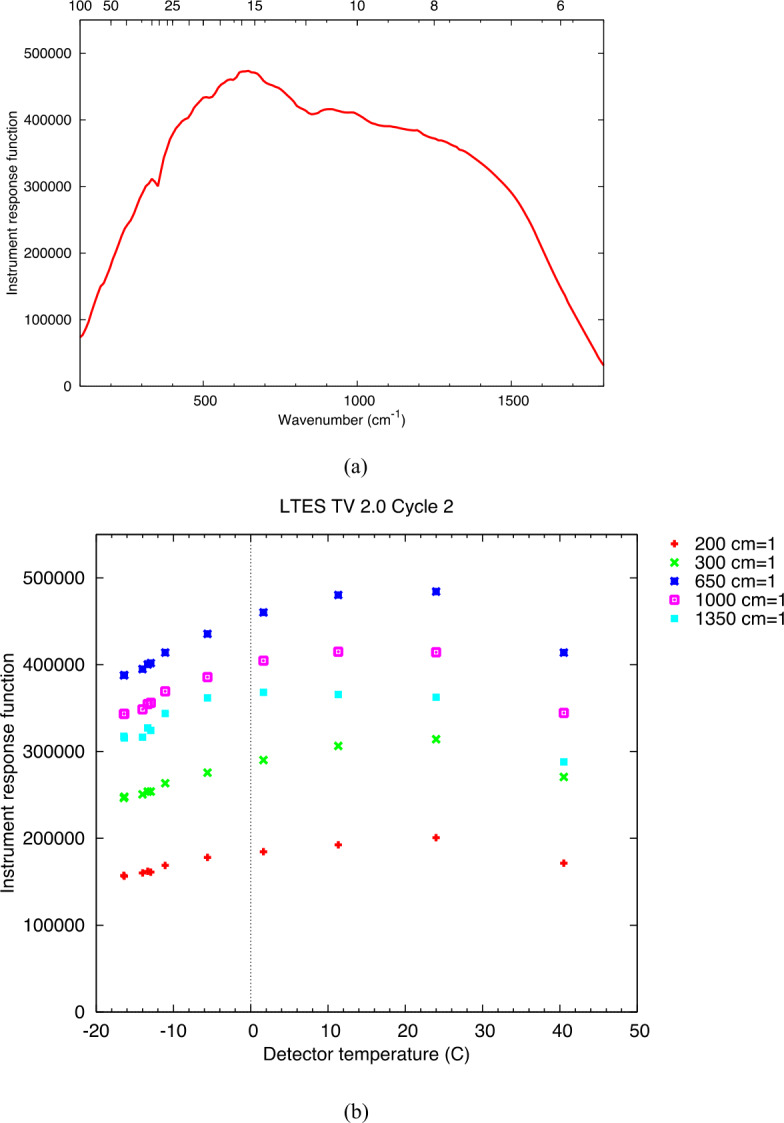
(a) The L’TES Instrument Response Function (IRF in units of Volt/(W cm^−2^ /cm^−1^ sr^−1^) for an instrument temperature of 10 °C. Upper scale is micrometers. **(b)** The variation in the IRF (in units of Volt/(W cm^−2^ /cm^−1^ sr^−1^) with temperature at five representative wavenumbers. These data were collected at the different instrument temperatures and target temperatures during thermal vacuum radiometric testing

The L’TES Instrument Response Function (IRF) provides the transfer from photons coming into the instrument to the output signal (voltage) from the instrument as an interferogram. The IRF varies with wavenumber due to the wavelength variations of the detector response, the diamond beamsplitter and lens transmission, and all the beamsplitter and mirror finishes and coatings. The IRF was determined as a function of instrument temperature through a two-point calibration using one of the BCU calibration blackbody standards together with the internal calibration blackbody during the thermal vacuum radiometric calibration. Figure 10a gives an example of the IRF for an instrument temperature of 10 °C, at the original servo velocity and IR sampling frequency of 656 Hz. The variation of IRF with instrument temperature for a representative set of wavenumbers is given in Fig. 10b. The primary cause of variation in response function is the variation in detector performance with temperature, and to a lesser extent, changes in the interferometer alignment with temperature. Over the L’TES in-specification operational temperature range of −10 to 30 °C and the performance spectral range of 300 to 1350 cm^−1^, the L’TES IRF varies by less than 15%.

The data in Fig. 10 were collected during pre-delivery thermal vacuum testing at ASU with the original IR sampling frequency of 656 Hz. Unfortunately, no precision radiometric data could be collected with the flight unit after the IR sampling frequency was increased to 772 Hz (see Sect. 5.1). The sampling frequency of the OTES instrument was also changed after thermal vacuum calibration (Christensen et al. 2018), and in-flight experience with OTES confirms that the IRF spectral performance is essentially identical between the original 656 Hz and final 772 Hz sampling frequencies. Therefore, we conclude that the IRF data presented in Fig. 10 is the performance that is expected for L’TES in flight. It is important to note that the IRF will be directly measured during all flight operations using observations of space and the internal calibration blackbody, and the radiometric calibration does not rely on the pre-launch IRF data.

### Spectral Sample Position and Spectral Range

The L’TES spectral sample position is a function of the total Michelson mirror displacement, which is determined by the metrology laser wavelength and the number of samples that were collected in each interferogram. During ambient testing atmospheric CO_2_ was observed and the L’TES spectrum was fit to a line-by-line modeled CO_2_ spectrum. The results from this test found the best-fit value for the laser wavelength to be 0.851 μm. Over the full expected L’TES operational temperature, the laser wavelength only varies by $<\pm0.17$%, which results in the L’TES IR sample spacing varying by $<\pm0.015$ cm^−1^. Based on this small error the laser wavelength will be assumed to be constant over temperature.

### Precision: Noise Equivalent Spectral Radiance

The L’TES precision, or noise equivalent spectral radiance (NESR), was determined by calculating a scene radiance using the two precision BCU calibration targets as outlined in Eq. (3). The NESR is defined as the standard deviation of the scene radiance and was determined during thermal vacuum testing operational instrument temperatures of −10, 0, 10, and 25 °C. In addition to the intrinsic noise in the system, any changes in the temperature of the target or the instrument during the radiometric test will result in changes in the derived scene radiance, so that the measured NESR is the upper limit on the true system performance. To minimize these additional noise sources, the L’TES instrument and the BCU targets were maintained at as stable a temperature as possible and the observations of each target were limited to 200 s.

**Fig. 11:**
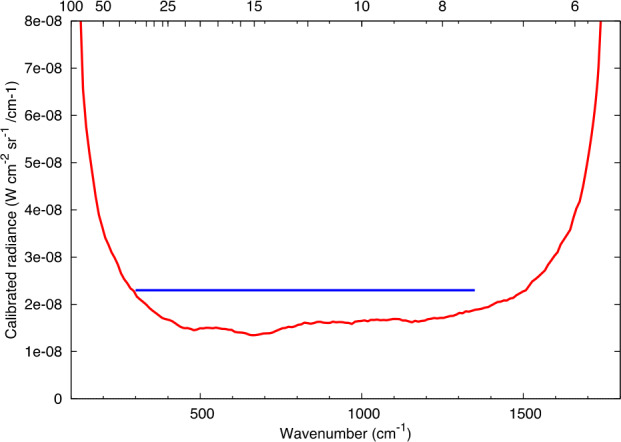
The L’TES noise equivalent delta radiance (NESR in units of W cm^−2^ /cm^−1^ sr^−1^), which represents the 1-sigma variation in calibrated radiance. These data were collected in thermal vacuum radiometric testing at the original moving mirror sampling frequency of 656 Hz

The thermal vacuum NESR results are given in Fig. 11. These data were collected during pre-delivery thermal vacuum testing at ASU with the original moving mirror velocity and an IR sampling frequency of 656 Hz.

At the original sampling frequency L’TES meets the performance requirement over the full range from 300 to 1350 cm^−1^ (Fig. 11). However, as discussed in Sect. 5.1, the moving mirror velocity and IR sampling frequency were increased to move the observed microphonic interference from the IMU out of the L’TES information band. Unfortunately, a complete and systematic set of data could not be collected at LMC to determine the L’TES NESR after the sampling velocity was increased. The L’TES instrument performance model predicts a 20% increase in the NESR at the higher servo velocity, but half of this increase will be recovered due to the larger number of spectral bands. Because the primary science objective is to determine the asteroid temperature by fitting a set of blackbody curves to the measured spectrum, the slightly higher NESR will have an insignificant effect on the primary L’TES science objective.

### Absolute Radiometric Calibration

**Fig. 12:**
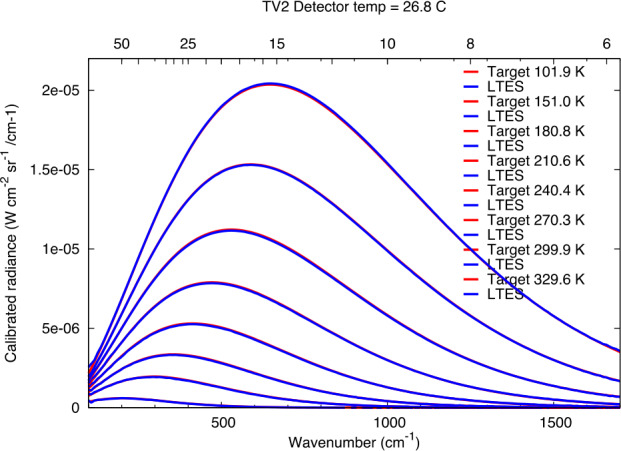
The calibrated radiance computed by L’TES during thermal vacuum testing. The data were collected at an instrument (detector) temperature of 26.8 °C viewing the BCU target whose temperature was varied from ∼100 to ∼300 K. The blue curved are the L’TES-derived radiances; the red curves are the computed Planck emission

During the L’TES thermal vacuum testing the calibrated radiance was computed using Eq. (3) through observations of one of the NIST-calibrated BCU targets, whose temperature was set to 85 K to simulate space observations, together with observations of the internal cal target. The second BCU was treated as the “scene”, and its temperature was varied from 100 K to 330 K to simulate the full range of expected scene temperatures that will be observed by L’TES in flight. In deriving the calibrated radiance, it was assumed the emissivities of both BCU targets and the internal cal target were exactly 1.0, and that the temperatures of the targets were accurately given by their thermistor measurements. The results are shown in Fig. 12, which gives both the L’TES-derived calibrated radiance and the computed Planck emission. As can be seen the L’TES absolute calibration is excellent.

**Fig. 13:**
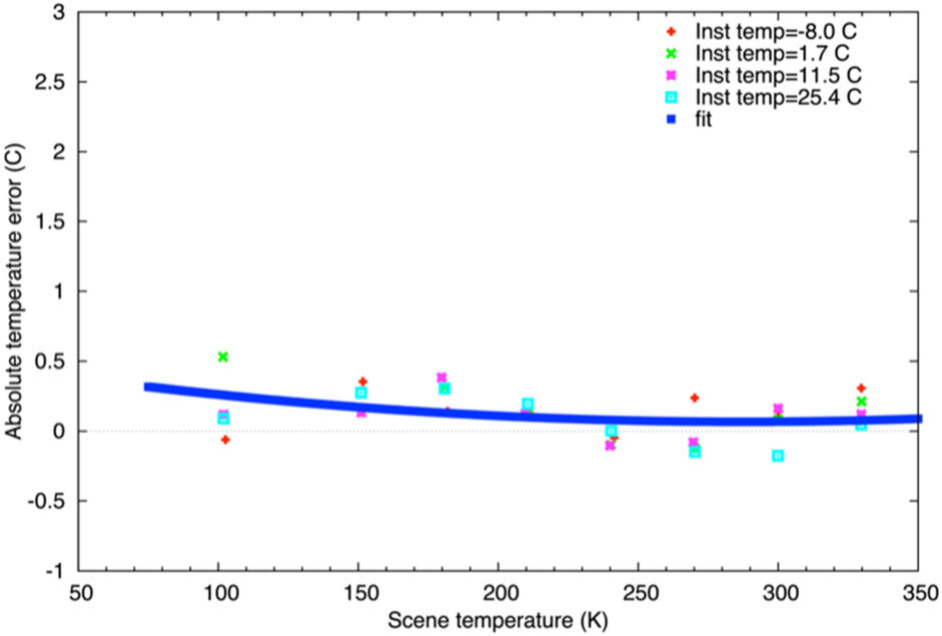
The measured absolute temperature error. The calibrated radiance was computed for each target temperature at each instrument temperature and the kinetic temperature was derived. The absolute temperature error is the difference between this temperature and the measured temperature of the BCU target (see text for details)

The L’TES absolute temperature requirement is 2 K for target temperature ≥75 K (Sect. 2.2). The absolute accuracy is controlled by the knowledge of each of the parameters in Eq. (3), with the primary error sources being the calibration blackbody temperature and emissivity (Christensen et al. 2018). The absolute temperature performance was verified by using the calibrated radiance (Fig. 12) to compute a brightness temperature at each wavenumber assuming unit emissivity/ The resulting brightness temperature spectrum was filtered to reduce noise effects and the maximum of the brightness temperature was then assumed to be the kinetic temperature of the BCU target. Figure 13 shows the results for each of the eight scene temperatures acquired at each of the four instrument temperatures (Sect. 4.1) and confirms that L’TES readily meets its 2 K absolute temperature requirement.

## L’TES Mission Operations

### In-Flight Operation

L’TES is operated using spacecraft-commanded sequences that consist of power on, a warm-up and thermal stabilization period, a series of alternating observations of the external scene and the internal calibration blackbody, and power off. Upon receipt of power the L’TES moving mirror begins operating in the default 2-sec scan mode and L’TES begins collecting and outputting telemetry information. In this “stand-by” mode, no interferogram data are output. An L’TES command from the spacecraft puts the instrument into its interferogram output “science” mode. In the science mode L’TES collects groups of interferograms viewing the external scene with the calibration flag open, and groups of interferograms viewing the internal calibration blackbody with the calibration flag closed. During each asteroid encounter L’TES will begin viewing the asteroid as an unresolved point source. Periodic slews of the IPP away from the asteroid will provide L’TES with space observations, and periodic observations will be obtained of the internal cal target. The timing of these observations will be determined from in-flight experience, with the goal of acquiring observations at the appropriate intervals to adequately remove the L’TES temperature variations while minimizing movement of the IPP and interruptions of the asteroid observations. During closest approach the observations of the internal cal target will be minimized to maximize the asteroid science data collection. If no space views are available, then a previously determined Instrument Response Function will be used.

### In-Flight Calibration and Observations

The L’TES instrument data will be calibrated in flight using Eq. (3), together with periodic views of space and the internal calibration blackbody. Typical operation will collect 10 interferograms of the internal calibration blackbody and ∼10 interferograms of space when possible. All interferograms are converted to spectra through a discrete Fourier transform that is performed on the ground. Following this transform, the spectra in each group of space and cal observations will be averaged and then interpolated between successive space or calibration observations. If only a single group of space or cal observations are collected, these will be used for the entire sequence. If science spectra are collected before or after the first or last space or cal observation, these bounding space or cal observations will be extended forward or backward in time as appropriate. If only calibration blackbody or space observations are acquired in a sequence, then the pre-launch IRF, at the appropriate instrument temperature, will be used with the available calibration observation determine the scene radiance.

In-flight experience with the TES and OTES instruments demonstrated that the instrument response function varied by less than 5% over the life of those missions (Christensen et al. 2001), and a similar stability is expected for the L’TES instrument. The calibration requires knowledge of the instrument radiance ($I$_detector_), which is determined from observations of space and the cal target. Because the instrument temperature will vary slightly over time, periodic observations of space and the cal target are required, and the instrument radiance used for each scene spectrum is assumed to be a linear interpolation of the values derived from each bounding set of calibration observations.

**Fig. 14:**
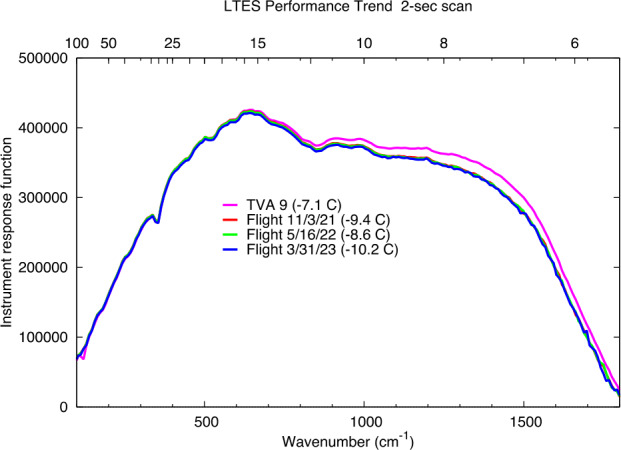
The L’TES Instrument Response Function. All of the data were collected at an instrument temperature of approximately −10 °C and confirm that the L’TES performance remains constant in space and is very close to the performance measured in thermal vacuum (TVA 9)

**Fig. 15:**
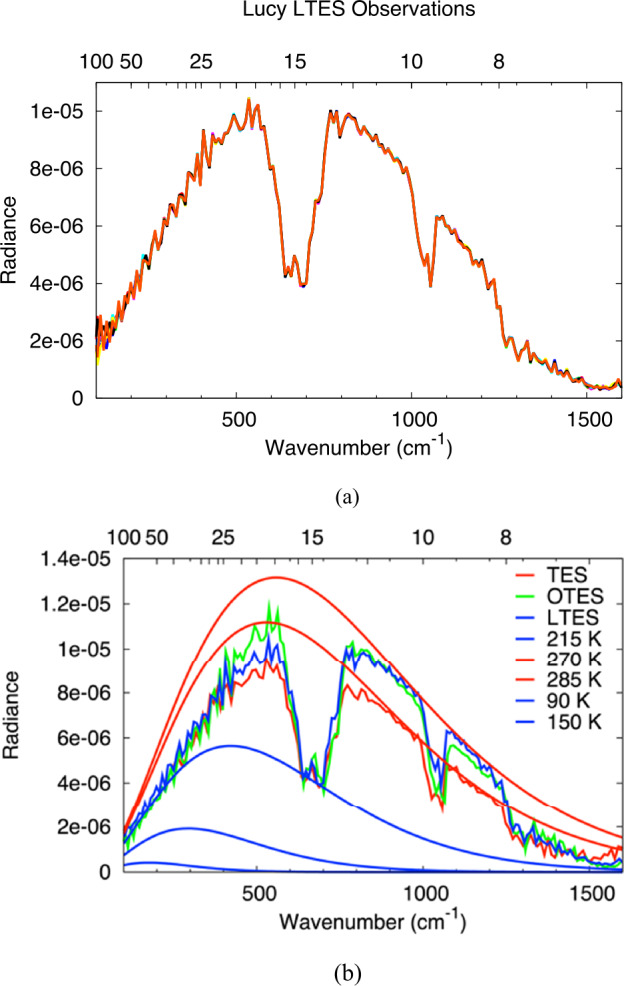
L’TES observations of the Earth. a) Ten consecutive TES spectral collected at 10 cm^−1^ spectral resolution during the Lucy Earth gravity assist flyby in Oct. 2022. The spectral signatures are due to gases in the Earth’s atmosphere and their repeatability between spectra confirms the excellent L’TES noise performance

As of May 2023 L’TES had been turned on three times in space for instrument checkout and once to collect Earth-Moon observations. The three checkouts were acquired at instrument temperatures close to −10 °C and the derived IRFs are essentially identical and close to the IRF measured prior to instrument delivery at a temperature of −7 °C (Fig. 14). The slight change in IRF post-launch is due to the expected slight change in the interferometer alignment once it is operating in a zero-G environment. L’TES observations of the Earth were acquired during the Lucy Earth gravity assist flyby on Oct. 13, 2022. Ten consecutive spectra acquired at 10 cm^−1^ resolution are shown in Fig. 15a. The spectral features observed are due to water vapor, CO_2_, and methane and the consistency of these spectra is an excellent indicator of the low spectral noise that is achieved with L’TES. Figure 15b shows a comparison of similar observations acquired in 1996 by the Mars Global Surveyor TES instrument (Christensen and Pearl 1997) and in 2017 by the OSIRIS-REx OTES instrument (Christensen et al. 2018). The radiance differences in the 8-14 μm atmospheric windows are due to the differences in ocean and land surface temperatures.

## Data Processing and Archiving

The L’TES data processing pipeline is straightforward and based on the flight-proven OTES and methodology and software (Christensen et al. 2018). Each L’TES sequence, from power on to power off, will be processed independently, and the interferograms are returned and processed separately on the ground in the forward and reverse scan directions. Observations of space will be identified using the spacecraft pointing data. Each interferogram will be transformed to a spectrum and processed to a calibrated radiance spectrum. Bad or noisy spectra will be identified and removed prior to averaging groups of space or cal target data. The temperatures of the internal blackbody and optical elements will be determined using the redundant thermistor data, with testing done to eliminate erroneous data. These data provide the inputs to Eq. (3) that are used to compute the calibrated radiance for each scene spectrum.

The L’TES standard data product is calibrated spectral radiance. These data, along with all of data used in the calibration, will be delivered to the Planetary Data System on the schedule agreed to by the Lucy Project. For each spectrum the calibrated radiance is converted to brightness temperature at each wavenumber, assuming unity emissivity. The processing continues to compute surface temperature in a full emissivity-temperature separation process. Temperature uncertainties are computed for use in the thermal inertia error analyses. These uncertainties are computed using an initial estimate of the surface temperature from the brightness temperature to constrain the temperature-dependent wavenumber range over which to average the brightness temperature, computing a new surface temperature, and repeating until the results converge.

## Abbreviations

ADC: analog to digital converter
ARM: antireflection microstructure
ASU: Arizona State University
BCU: Bench Checkout Units
CDR: Critical Design Review
CPT: Comprehensive Performance Test
CVD: chemical vapor deposited
DFT: discrete Fourier transform
DLATGS: deuterated l-alanine doped triglycine sulfate
FET: Field Effect Transistor
FPGA: Field Programmable Gate Array
FPU: Field Programmable Unit
FWHM: full-width, half-maximum
GSE: Ground Support Equipment
IFOV: instantaneous field of view
IFT: Instrument Functional Test
IMU: inertial measure unit
IPP: Instrument Pointing Platform
IRF: instrument response function
LMC: Lockheed Martin Corporation
L’TES: Lucy Thermal Emission Spectrometer
MER: Mars Exploration Rover
MGS: Mars Global Surveyor
Mini-TES: Miniature TES
MLI: Multi-Layer Insulation
MO: Mars Observer
NE$\Delta \varepsilon $: Noise Equivalent Delta Emissivity
NESR: noise equivalent spectral radiance
OTES: OSIRIS-REx Thermal Emission Spectrometer
PDR: Preliminary Design Review
S/C: spacecraft
SRR: System Requirement Review
TES: Thermal Emission Spectrometer
THEMIS: Thermal Emission Imaging System
ZPD: zero path difference
